# Post-harvest practices for aflatoxin control: Evidence from Kenya

**DOI:** 10.1016/j.jspr.2019.03.001

**Published:** 2019-06

**Authors:** Alexia Pretari, Vivian Hoffmann, Lulu Tian

**Affiliations:** a International Food Policy Research Institute, 1201 Eye St NW, Washington, DC, 20005, USA; b Innovations for Poverty Action, P.O. Box 72427-00200, Nairobi, Kenya

**Keywords:** Aflatoxin, Post-harvest technologies, RCT, Maize, Kenya

## Abstract

We assess the impact of a package of post-harvest technologies on aflatoxin contamination of maize through a randomized trial in rural Kenya. Some elements of this package (training and provision of plastic sheets for sun-drying) were provided free of charge to all participants in treatment villages and were widely adopted. Others (a mobile drying service and hermetic storage bags) were provided free to a subset of randomly selected farmers in treatment villages while others had to pay. Overall, the intervention reduced aflatoxin contamination by over 50%. Most of this reduction appears to be due training and the use of drying sheets, the lowest-cost of all the technologies offered.

## Highlights

• Training and access to post-harvest technologies were offered to maize farmers in Kenya.
• Aflatoxin and moisture content were measured in stored maize 3 months later.
• The intervention reduced aflatoxin contamination in stored maize by 53%.
• Training and use of drying sheets appear to drive this reduction.

## 1 Introduction

Exposure to aflatoxin, a common fungal toxin, is associated with a number of negative health consequences. Acute exposure can cause hepatitis, liver failure, and death (Strosnider et al., 2006). Multiple outbreaks of aflatoxicosis in Kenya have been reported since 2005, when 125 people died from consuming highly contaminated home-grown maize (Daniel et al., 2011). An association between chronic aflatoxin exposure and liver cancer is well documented, and the toxin is classified by the International Agency for Research on Cancer as a Group 1 carcinogen. Emerging evidence also suggests possible detrimental effects on child growth (Gong et al., 2004; Strosnider et al., 2006; Turner et al., 2007; Hoffmann et al., 2018).

Aflatoxin exposure is high in many developing countries, where testing and regulatory capacity are low, environmental factors facilitate fungal colonization and growth, and post-harvest handling and storage practices are often poor (Gnonlonfin et al., 2013). The importance of post-harvest practices for aflatoxin control in maize has been demonstrated in observational studies (Hell et al., 2000; Udoh et al., 2000). Experimental work has shown that both rapid drying of maize and storage of dry maize in hermetic bags dramatically reduce aflatoxin levels (Kaaya and Kyamuhangire, 2010; Williams et al., 2014; Ng'ang'a et al., 2016). Preventing contact between harvested crops and soil, a reservoir of the fungus that produces aflatoxin, is also recommended (Hell and Mutegi, 2011).

We evaluate the impact of a package of post-harvest technologies appropriate for use by smallholder farmers on contamination of maize with aflatoxin. Impacts of the intervention on reported post-harvest losses are also assessed. Our study builds on previous work showing the importance of post-harvest practices to aflatoxin control, most significantly a trial conducted in the Gambia, which showed that providing farmers with training and a package of simple post-harvest technologies significantly reduced aflatoxin contamination of stored peanuts as well as human exposure to the toxin (Turner et al., 2005).

## 2 Methods

### 2.1 Sample selection and baseline survey

The study sample consists of maize farmers in 30 randomly selected maize-growing villages in Meru and Tharaka-Nithi counties in Kenya, a region where aflatoxin contamination of maize leads to frequent and sometimes fatal outbreaks of aflatoxicosis. Each of the 30 study villages was visited in June 2013 for baseline survey data collection. A total of 679 households across these villages, each of which included either a pregnant woman or child under 24 months of age, were enrolled in the study. Each enrolled household was administered a structured questionnaire that included modules on household composition, food and other consumption, and farm practices.

### 2.2 Randomized intervention

To provide some level of benefit to all study villages, basic training on aflatoxin prevention was provided to all study villages through a training-of-trainers approach in July 2013. At least one farmer from each of the study villages was selected in consultation with community leaders to receive basic training on the health consequences and prevention of aflatoxin contamination in maize. Trained farmers were asked to pass on this information to others in their communities, and provided compensation based on their reports and confirmation by others that they had done so.

In addition to this basic level of training, 15 of the study villages were randomly assigned to a *technology treatment* group. Farmers in the remaining 15 villages constituted the control group. Maize farming households in *technology treatment* villages were visited at their homes and invited to attend an information meeting about aflatoxin prevention. Farmers were asked how much maize they expected to harvest during the current season. They were informed that plastic sheets for sun drying of maize would be provided to all attendees, and that those who attended would also have an opportunity to access a maize dryer and hermetic storage bags, potentially for free or at a discounted price. A flyer containing information about the meeting's purpose, time and location was left with all invitees.

At the meetings, study staff described post-harvest practices for aflatoxin prevention. These included the prevention of contact between maize and the ground during drying, shelling techniques to avoid damage to kernels, sufficient drying, and storage in clean bags raised off the ground. The maize drying service offered through the study was then described. A photograph of the flatbed dryer was shown, and a hermetic storage bag was passed around for farmers to examine. The mobile dryer used was a prototype of the EasyDry500, designed by ACDI-VOCA (http://www.acdivoca.org/easydry/). This dryer is similar in design to the biomass dryer described by Mwema et al. (2015), except that the drying chamber consists of a single bed rather than a vertical chimney.

A lottery was then conducted to determine the price at which the maize dryer and hermetic storage bags would be offered to each study participant. Hermetically sealable Purdue Improved Cowpea Storage (PICS) storage bags were locally available at a price of 220 Kenyan Shillings (KSh). The maize dryer was not commercially available, but based on equipment, capital, and labor costs, we estimated the that a for-profit drying service would charge 350 KSh per 90 kg bag of maize. Meeting participants in *technology treatment* villages were randomly assigned to one of three price treatments:

1. *Full price* (50% probability): The mobile dryer could be used at a price of 350 KSh/bag (3.89 KSh/kg) and hermetic bags could be purchased at 220 KSh each.
2. *Partial subsidy* (25% probability): The mobile dryer could be used at a price of 150 KSh/bag (1.67 KSh/kg) and hermetic bags could be purchased at 95 KSh each.
3. *Full subsidy* (25% of probability): The mobile dryer could be used free of charge. One hermetic bag, plus one additional bag for every two bags of maize produced, was provided free of charge. Additional bags could be purchased at the full price of 220 KSh.

Farmers within *technology treatment* villages were randomly assigned to a *market incentive* treatment, under which they would a premium price for maize found to conform with the national aflatoxin standard 3 months after harvest. The market incentive treatment is further described in Hoffmann and Jones (2018). Meeting participants were given a booklet written in Kiswahili and illustrated with simple graphics that described recommended post-harvest practices for aflatoxin control including information on how to access the dryer. Plastic sheeting (500 gauge) measuring 2.5 m by 6 m, available locally at a cost of approximately 5 USD per piece, was provided to all meeting participants free of charge.

Information on participants' expected dryer use was collected at the meeting and appointments with dryer operators were later finalized by phone. The drying service included transportation of the dryer to the farm or nearby site (in which case moving the maize to the dryer location was also included), measurement of initial grain moisture content using a hand-held SuperTech Agroline Superpro moisture meter according to manufacturer instructions, and use of the flatbed dryer until a moisture content of 13.5% was achieved. To prevent participants assigned to the higher price treatment groups from accessing the dryer at the free or discounted price, farmers were allowed to dry only as much maize as they had previously stated they expected to harvest. The number of hermetic bags they could purchase was similarly limited based on expected harvest amount. Participants’ maize was weighed and participants had to pay upfront the cost for drying the maize. Those whose maize tested at or below 13.5% moisture prior to drying were refunded the fee, and drying was not performed on their maize. Participants could then purchase or obtain for free (depending on the price treatment to which they were assigned), hermetic bags to store up to the amount of maize that had been dried using the mobile dryer, or had been tested and found to already be sufficiently dry. Restricting hermetic bag access in this way was intended to avoid exacerbating aflatoxin risk and spoilage of maize in general. After the maize was dried, the research team demonstrated how to utilize and securely close the hermetic bag.

Table 1 shows rates of meeting participation, receipt and usage of technologies offered among farmers assigned to the *technology treatment* group. The vast majority of households attended the training meeting (93%) and received plastic sheeting (92%). A third of meeting participants (*N* = 114) did not take part in the lottery to determine the price at which the mobile dryer and hermetic bags could be accessed. This was primarily because farmers expected a harvest below the 45 kg minimum required to use the dryer (*N* = 99). Farmers generally prefer not to mix their maize with that produced by others and partitioning the dryer bed to deal with volumes below 45 kg was impractical. Fifteen attendees refused from other reasons, for example discomfort with gambling.

**Table 1:** Participation in intervention activities and take-up of technologies (technology treatment group only): means ± standard error.

Variable	Mean ± *SE*	Observations
Attended meeting	0.93 ± 0.01	350
Received plastic drying sheet	0.92 ± 0.01	350
Did not take part in the lottery	0.33 ± 0.03	350
Drew full discount (free) token	0.35 ± 0.03	350
Drew partial discount token	0.17 ± 0.02	350
Drew full price token	0.15 ± 0.02	350
Presented payment for dryer at assigned price	0.38 ± 0.03	350
Dried any maize (maize had MC > 13.5%)	0.12 ± 0.02	350
Obtained at least one hermetic bag (free or purchased)	0.34 ± 0.03	350
Number of hermetic bags obtained	0.45 ± 0.04	350

Just over half (56%) of households who drew a price brought maize to the dryer, and sufficient cash to cover their assigned cost of drying. As expected, demand for the dryer was highest in the full discount group, and lowest in the full price group (these results, as well as the impact on dryer demand of a premium price for aflatoxin-safe maize, are described in detail in Hoffmann and Jones (2018)). However, because much of the maize brought for drying was already below 13.5% moisture content, only 12% of those in the treatment group actually had their maize dried by the dryer. Thirty-four percent of households in treatment villages obtained one or more hermetic bags. Most of these households obtained just one bag, and the average number of hermetic storage bags among those who obtained any through the study was 1.3.

### 2.3 Follow-up data collection

Researchers visited households approximately 3 months after harvest to sample stored maize and administer a brief survey concerning maize drying and storage practices. Any home-grown maize that the household (a) planned to consume within the next two weeks, or (b) planned to sell, was identified for sampling. Lots were defined as maize stored in a particular bag or other storage container. If a household had several lots of home-produced maize dedicated to future sale, these were numbered and a randomization table was used to identify the lot of maize from which to sample maize. From each lot of maize identified for sampling, enumerators were trained to pierce the storage container, which was usually a woven polypropylene bag, from the top, middle and bottom, and from opposite sides using a sampling spear. From each of six samples obtained in this way, at least 50 g of maize grain were taken and homogenized. Moisture content was measured using a subsample of the homogenized maize. The same homogenized sample was then milled, weighed and passed through a 20-mesh sieve. Per GIPSA sampling guidelines (GIPSA, 2000), the portion of the sample that passed through this sieve was weighed; if the proportion of the sample that passed through the sieve was less than 70%, it was ground again until at least 70% passed through. Out of the ground and sifted mix, 50 g was taken to perform the test. Maize samples were tested on site using the Neogen Q+ test and converted to quantitative values up to 111 parts per billion (ppb) using Mobile Assay's mReader. Three readings were taken within one minute for each sample, and the average reading over the three is used in the analysis presented in Section 3. For ethical reasons, any lots of maize from which samples tested over the regulatory limit of 10 ppb total aflatoxins were immediately swapped, with the participant's consent, for maize that had tested below this level. Approximately one and a half months later, households were visited again and administered an endline household survey similar in structure and content to the baseline survey.

### 2.4 Statistical analysis

Analysis of the intervention's impact was conducted using multivariate linear regression based on an intent-to-treat approach, meaning that households assigned to treatment villages are considered as having received the intervention regardless of their participation in intervention activities or their use of technologies offered through the study. Standard errors are bootstrapped to correct for clustering at the village level for all estimations. As adoption of new post-harvest practices and agronomic practices more generally may be affected by household wealth, harvest size, and market engagement, we include controls for monthly consumption value per adult equivalent, household size, total maize harvest, and whether the household sold any maize over the year preceding baseline. In addition, we include as controls any variables for which the mean baseline value differs between experimental groups either in the full sample, or among those with maize in store during the maize collection survey, as described in section 3. In case of missing value of a given control variable, the observation is included in the analysis (the average value of the variable is attributed to this observation) and an indicator variable is included to account for this imputation. Out of 679 observations in the whole sample, 13 have at least one control variable with a missing value; out of 179 observations of households with home-produced maize in store, 4 have at least one control variable with a missing value. Note that the analysis of the intervention's impact based on an intent-to-treat approach was also conducted using linear regressions without control variables and that the results presented in section 3.2 are consistent in the absence of controls.

We also report non-experimental evidence on the efficacy for aflatoxin control of the three technologies offered. Because the number of maize samples collected from farmers who used some combinations of technologies used is limited, we present means and confidence intervals for each category of use, and do not adjust these values for baseline characteristics, nor for the clustered structure of the data. Throughout the paper, statistical significance of the treatment effect or of differences in means is tested though two-tailed tests. Absolute *Z-*values or *t* statistics, and *P*-values are reported.

## 3 Results

### 3.1 Sample characteristics and balance across treatment groups

Results from the baseline survey indicate that households in treatment and control villages were generally similar. Group means and P-values of means tests for key variables are shown in Table 2. Households consisted of just over five members on average, and the mean age of the head was 37 years. Nearly 70% of household heads, 90% of whom were male, had completed primary school, but only 16% had completed secondary school, and fewer than 5% had any tertiary education. In addition to self-employment in farming, which was a condition of recruitment into the sample, 19% of household heads also worked as agricultural laborers. The mean monthly household expenditure per adult equivalent was approximately 3700 Kenyan shillings, around $37 US. Consumption per adult equivalent is similar to per capita consumption, except that members are assigned weights proportional to their caloric needs, based on age and sex. Livestock ownership is common in the sample, with the average household owning more than four chickens and two goats, sheep or pigs. 41% of households in the sample owned at least one large animal (cattle or horse), and the overall average number of cattle held was 0.6. Ownership of electronic or transportation assets (cell phones, televisions, VCRs, DVDs, bicycles and motor vehicles), on the other hand, is relatively low, at less than two on average. The farmers constituting the study sample owned on average less than two acres of land. During the main agricultural season, farmers planted 1.2 acres on average under maize. The mean annual harvest of maize was 539 kg over the two agricultural seasons preceding the baseline survey. The majority of the study sample (57%) cultivated maize solely for household consumption. Two thirds of respondents had heard of aflatoxin before, and 58% described aflatoxin as a mold and/or a toxin. While the importance of fully drying maize prior to storage was named by 85% of respondents as a way to prevent aflatoxin contamination, only a quarter mentioned preventing contact with the soil as an aflatoxin prevention strategy. Fewer respondents (59% versus 73%, *Z* = 2.75, *N* = 673, *P = *0.01) in treatment villages had heard of aflatoxin at baseline compared to those in control areas. In addition, households in treatment villages owned slightly fewer poultry (5.3 vs. 4.1 birds on average, *Z* = 1.97, *N* = 679, *P* = 0.05).

**Table 2:** Comparison of household characteristics, by experimental arm: means ± standard error, and test of the difference between means.

Variable	Mean ± *SE* in control villages	Mean ± *SE* in treatment villages	|*Z*-value| of means test	*P*-value of means test	Observations
Household size	5.1 ± 0.10	5.1 ± 0.10	0.01	(0.99)	679
Head is a female	0.09 ± 0.02	0.12 ± 0.02	0.97	(0.33)	679
Head's age	37 ± 0.61	37 ± 0.64	0.43	(0.67)	679
Head completed primary school	0.70 ± 0.03	0.67 ± 0.03	0.44	(0.66)	678
Head completed secondary	0.17 ± 0.02	0.15 ± 0.02	0.41	(0.68)	678
Head employed as farm labourer in the past	0.17 ± 0.02	0.21 ± 0.02	0.86	(0.39)	678
Month					
Monthly consumption expenditure per adult	3648 ± 150	3709 ± 137	0.14	(0.89)	678
equivalent (KSh)					
Number of non-animal assets	1.8 ± 0.09	1.7 ± 0.08	0.21	(0.84)	679
Number of poultry owned	5.3 ± 0.33	4.1 ± 0.27	1.97	(0.05)	679
Number of goats, sheep, pigs	2.1 ± 0.16	1.8 ± 0.14	0.77	(0.44)	679
Number of horses and cattle	0.6 ± 0.06	0.7 ± 0.06	1.00	(0.32)	679
Acres land owned	1.9 ± 0.10	1.6 ± 0.09	0.89	(0.37)	679
Acres under maize main season	1.3 ± 0.06	1.2 ± 0.06	1.16	(0.25)	612
Total maize harvest during both seasons (kg)	573 ± 36	567 ± 49	0.04	(0.97)	642
Sold any maize past year	0.5 ± 0.03	0.38 ± 0.03	0.95	(0.34)	668
Heard of aflatoxin	0.73 ± 0.02	0.59 ± 0.03	2.75	(0.01)	673
Describes aflatoxin as a mold	0.46 ± 0.03	0.38 ± 0.03	1.85	(0.06)	673
Describes aflatoxin as a toxin	0.08 ± 0.01	0.05 ± 0.01	1.36	(0.17)	673
Describes aflatoxin as both mold and toxin	0.11 ± 0.02	0.09 ± 0.02	0.75	(0.45)	673
Drying maize well before storage prevents	0.87 ± 0.02	0.83 ± 0.02	1.49	(0.14)	673
Aflatoxin					
Drying maize off bare ground prevents	0.27 ± 0.02	0.22 ± 0.02	1.08	(0.28)	673
aflatoxin					

Notes: Non-animal assets are electronic items (TV, video/DVD/VCR, mobile phone) and transportation assets (bicycle, motorcycle, car/truck/tractor). Standard errors in means tests are bootstrapped and clustered at the village level cluster.

Since analysis of aflatoxin contamination is restricted to households with home-produced maize in store during the maize collection survey, we also test for baseline balance within this sub-sample (table not shown). We find that the number of large livestock (cattle or horses) owned differ significantly across experimental treatment groups within the sample (0.5 vs 0.9, *Z* = 1.76, *N* = 179, *P* = 0.08). The proportion of household heads who engaged in paid agricultural laborer also differ across the two groups, the statistical significance of the difference being very close to the 10% threshold (12% vs 17%, *Z* = 1.62, *N* = 178, *P* = 0.11). These four variables are included as controls in the analysis of the intervention's impact presented in Section 3.2. As presented in Table 2, 46% of respondents in treatment villages described aflatoxin as a mold, vs 38% in the control areas (*N* = 673, *Z* = 1.85, *P = *0.06), but this difference is driven by the difference in whether respondents have heard of aflatoxin before, hence this variable will not be included as a control variable. Similarly, among households with home-produced maize in store during the maize collection survey, 15% of respondents in treatment villages described aflatoxin as both a mold and a toxin, vs 9% in the control areas (*N* = 177, *Z* = 1.70, *P = *0.09), but this is also driven by the difference in whether respondents have heard of aflatoxin before (table not shown).

Data from the maize collection survey indicates that 68% of households kept their maize in a room in the main house or (sometimes separate) kitchen, which was also used for other purposes. Most of the remainder (31% overall) stored maize in a dedicated room in the house, while just 4.1% used a separate structure with roof and wall and 1.6% had a traditional granary with partially open walls. A handful of respondents had multiple storage facilities, but the vast majority reported keeping stored grain in a single storage area. While these data were collected post-intervention, we do not expect the intervention would have affected households’ storage structures, given the timing of the training immediately prior to harvest and the fact recommendations on storage structure were not given, and we find no differences in these variables across treatment groups.

### 3.2 Impact of the randomized intervention

Three months after harvest, households assigned to the treatment and control groups were similarly likely to participate in the maize collection survey (Table 3, column 1) and to have any home-produced maize in store (column 2). As much of the analysis below is performed on the subset of households with home-produced maize in store at time of the maize collection survey (just 179 of the 571 surveyed), we present hereafter the main differences, in terms of baseline characteristics, between these households and those without maize in store at the time. Households with maize in store at the time of the maize collection survey were smaller (4.7 vs 5.4 household members, *N* = 579, *Z* = 4.67, *P = *0.00), the head was more likely to be educated (21% completed secondary school, vs 12%, *Z* = 3.34, *N* = 578, *P = *0.00), they were better-off (higher per adult equivalent monthly consumption (*N* = 579, *Z* = 4.99, *P* = 0.00) and higher number of non-animal assets (*N* = 579, *Z* = 4.21 *P* = 0.00)); these households were also producing more maize (*N* = 544, *Z* = 3.74, *P* = 0.00), more likely to sell any (*N* = 570, *Z* = 2.30 *P = *0.02), more likely to have heard of aflatoxin prior to baseline (*N* = 573, *Z* = 2.50, *P = *0.01) and more likely to agree that “Drying maize well before storage prevents aflatoxin” (*N* = 573, *Z* = 2.38 *P = *0.02). This selection and how it affects the validity of the findings of this paper is discussed in section 4.

**Table 3:** Estimated impact of the intervention on post-harvest practices based on multivariate linear regression.

	(1)	(2)	(3)	(4)	(5)	(6)
	Maize collection survey completed	The household has home-produced maize in store	Maize currently in store, among households with home-produced maize in store			
			Any dried on the ground	Any dried on plastic sheeting	Any dried using a drying service	Any stored in hermetic bag
Treatment	−0.01	−0.05	−0.17**	0.62***	0.17	0.49***
|*Z*-value|	0.39	0.59	2.27	6.07	1.59	11.22
*P*-value	(0.70)	(0.56)	(0.02)	(0.00)	(0.11)	(0.00)

Observations	679	579	179	179	179	179
R-squared	0.03	0.10	0.13	0.41	0.19	0.35
Mean ± *SE* in control villages	0.89 ± 0.02	0.35 ± 0.03	0.24 ± 0.04	0.13 ± 0.03	0.02 ± 0.01	0.02 ± 0.01

Notes: Household size, monthly expenditures per equivalent adult, harvest size, and whether the household sold maize in the year preceding the baseline survey, as well as baseline characteristics that differed across treatment groups as described in Section 3.1 are included as controls. Missing values of control variables are imputed, and dummies for the observations being missing are included as control. Standard errors in regressions are bootstrapped and clustered at the village level cluster; ***P < 0.01, **P < 0.05, *P < 0.1.

Impacts on reported post-harvest practices among the sample with any home-produced maize in store are reported in columns 3 through 6 of Table 3. We observe dramatic increases in reported use of plastic sheeting for drying of this maize (62 percentage points on a base of 13% in the control group) as well as for storage in hermetic bags (49 percentage points on a base of just 2%). Both of these effects are significant at *P* < 0.001 (*Z* = 6.07 and *Z* = 11.22 respectively, *N* = 179). Fewer farmers reported drying their crop directly on the bare earth (17 percentage points on a base of 24% in the control group, *P* = 0.02, *Z* = 2.27, *N* = 179), and more farmers reported using a drying service, although this result is not statistically different between the two groups but close to the 10% threshold (*P* = 0.11, *Z* = 1.59, *N* = 179). Note that these practices are not mutually exclusive – a given farmer could have sun-dried some maize on plastic sheets and some directly on the earth, and then used the mobile dryer to further dry down his or her maize. Among those who used the mobile dryer, the average proportion of the total maize harvest dried in this way was 61%.

Endline survey data collected five months after harvest yields results consistent with those based on the maize collection survey and sheds additional light on changes in drying practices (results estimated using same multivariate regression models as for other outcomes, not reported in tables). Conditional on sun-drying their maize before shelling it, survey respondents in the treatment group were 40 percentage points more likely to report doing so on plastic sheeting compared to those in the control group, of whom only 3% used such sheets (*N* = 422, *Z* = 10.41, *P* < 0.001). Respondents in the treatment group were also 27 percentage points more likely to dry unshelled maize on any barrier (vs. 57%; *N* = 422, *Z* = 4.01, *P < *0.001). Households in the treatment group were 22 percentage points more likely to dry their maize at all after shelling (vs. 55%, *N* = 492, *Z* = 2.39, *P = *0.02). Conditional on sun-drying their maize after shelling, those in the treatment group were 43 percentage points more likely to use plastic sheets for this (vs. 3%; *N* = 321, *Z* = 5.70, *P* < 0.001). Drying shelled maize on some kind of barrier (generally, woven nylon bags) was a near-universal practice, reported by 99% of respondents in the control group, and the intervention had a very small impact on this, significant at 10%.

Impacts on reported post-harvest losses to both pests and mold, based on data from the endline survey, are also evident. These results, presented in Table 4, are particularly strong for losses due to pests: taking the exponential of the coefficient on the randomized treatment assignment indicates a reduction of losses due to pests by 44%, and of losses to mold by 26% (*N* = 557, *Z* = 3.64, *P < *0.001 for losses due to pests, *Z* = 3.11 and *P < *0.001 for losses due to mold). An increment less than the minimum value observed in the data was added to zero-valued observations of losses and aflatoxin prior to log transformation to allow inclusion of these observations in the log-transformed models. Assignment to the *technology treatment* group reduces the proportion of households that report any loss to pests by slightly more than half, from 31% to 15% (*N* = 557, *Z* = 3.71, *P < *0.001), and reduces the proportion reporting any mold losses by more than half, from 11% to 3% (*N* = 57, *Z* = 3.55, *P < *0.001). Estimated impacts on the levels of each type of loss are similar, but sensitive to the exclusion of outliers. In regression 1, omitting 7 outliers from the specification (defined as mean + 3 standard deviations) reduces the effect size of treatment on losses due to pest, but increase significance (treatment coefficient: −3.19, Z = 2.37, N = 550, P < 0.001). For regression 4, omitting 11 outliers reduces the effect size and renders the effect statistically insignificant (treatment coefficient: −0.69, Z = 1.29, N = 446, P = 0.25).

**Table 4:** Estimated impact of the intervention on reported post-harvest losses based on multivariate linear regression.

	(1)	(2)	(3)	(4)	(5)	(6)
	Losses in storage that occurred after the most recent harvest					
	Quantity (kg) lost to pest	Log-quantity (kg) lost to pest	Any loss to pest	Quantity (kg) lost to mold	Log-quantity lost to mold	Any loss to mold
Treatment	−9.4*	−0.57***	−0.16***	−3.2***	−0.30***	−0.08***
|*Z*-value|	1.72	3.64	3.71	2.59	3.11	3.55
*P*-value	(0.09)	(0.00)	(0.00)	(0.01)	(0.00)	(0.00)

Observations	557	557	557	557	557	557
R-squared	0.08	0.05	0.06	0.05	0.04	0.05
Mean ± *SE* in control villages	13.5 ± 4.15	0.14 ± 0.11	0.31 ± 0.03	4.4 ± 1.12	−0.49 ± 0.08	0.11 ± 0.02

Notes: Household size, monthly expenditures per equivalent adult, harvest size, and whether the household sold maize in the year preceding the baseline survey, as well as baseline characteristics that differed across treatment groups as described in Section 3.1 are included as controls. Missing values of control variables are imputed, and dummies for the observations being missing are included as control. Standard errors in regressions are bootstrapped and clustered at the village level cluster; ***P < 0.01, **P < 0.05, *P < 0.1.

**Fig. 1:**
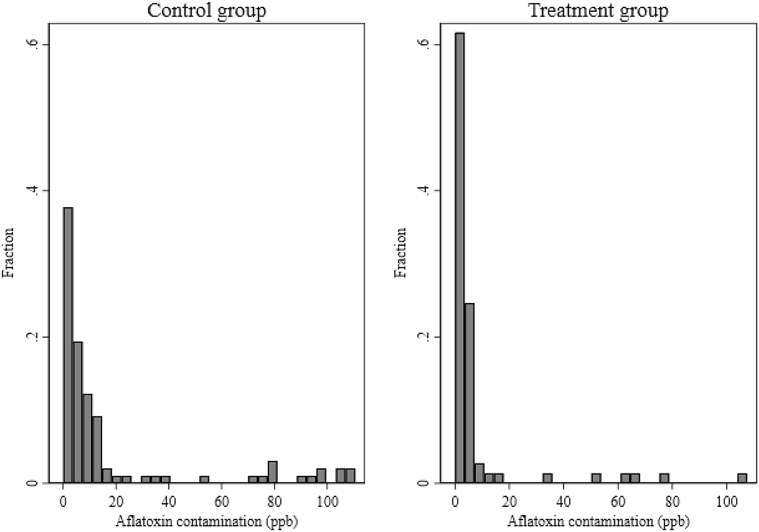
Distribution of aflatoxin level (ppb) in home-produced maize, by experimental arm.

We next turn to the impact of the intervention on aflatoxin contamination. For households with home-grown maize stored separately for sale and home consumption (*N* = 21), both types of maize were sampled and the mean aflatoxin value of these two samples is used in the analysis. For those with maize stored only for consumption (*N* = 150), this sample is used. As shown in Table 5 column 1, the intervention reduced aflatoxin contamination in stored maize by 9.9 ppb, 53% of the control group mean. The proportion of households with maize in store testing over 10 ppb, the regulatory limit, is hence drastically reduced (from 31% in the control group, to 9% in the treatment group, *N* = 171, *Z* = 3.17, *P* < 0.05). Analysis of the impact on log aflatoxin yields the same result, with the exponential transformation of the coefficient translating to a 53% reduction (*N* = 171, *Z* = 2.09, *P = *0.04). Moisture content in stored grain is also slightly lower for those in villages assigned to the intervention group (reduction of 0.54 on a basis of 14% moisture content on average in the control group, *N* = 171, *Z* = 1.81, *P* < 0.1). The distribution of aflatoxin contamination in maize samples by treatment group are plotted in Fig. 1, which shows a higher proportion of undetectable or very low values in the treatment group compared to the control, as well as fewer values at or near the upper limit of the test's range of detection.

**Table 5:** Estimated impact of the intervention on aflatoxin and moisture in stored, home-produced maize, based on multivariate regression.

	(1)	(2)	(3)	(4)
	Aflatoxin level (ppb)	Log-aflatoxin	Is over 10 ppb	Moisture content (%)
Treatment	−9.9***	−0.76**	−0.22**	−0.54*
|*Z*-value|	3.03	2.09	3.17	1.81
*P*-value	(0.00)	(0.04)	(0.00)	(0.07)

Observations	171	171	171	171
R-squared	0.05	0.06	0.11	0.16
Mean ± *SE* in control villages	18.5 ± 3.07	1.7 ± 0.18	0.31 ± 0.05	14.1 ± 0.13

Notes: Household size, monthly expenditures per equivalent adult, harvest size, and whether the household sold maize in the year preceding the baseline survey, as well as baseline characteristics that differed across treatment groups as described in Section 3.1 are included as controls. Missing values of control variables are imputed, and dummies for the observations being missing are included as control. Standard errors in regressions are bootstrapped and clustered at the village level cluster; ***P < 0.01, **P < 0.05, *P < 0.1.

For 8 households with home-produced maize in store, measurements of aflatoxin and moisture content are missing.

### 3.3 Results by technology use

Although all study farmers in villages assigned to the *technology treatment* were given plastic sheets and could access the maize dryer and hermetic storage bags, usage of these technologies was far from universal (Table 1). This was the case even for farmers assigned to the *full subsidy* sub-treatment. Usage required that the farmer had harvested at least 45 kg of maize, participated in the random price draw, and valued these technologies sufficiently to merit bringing maize to the dryer and (in the case of the partial subsidy or full price groups) paying the randomly assigned price. A further condition for use of the dryer, intended to prevent farmers from wasting money on an unnecessary service, was that maize had to have a moisture content above 13.5%, the recommended maximum for long-term storage. Only 31% of the maize brought to the dryer was above the 13.5% cutoff and thus dried. Of those who brought maize to the dryer, the majority (90%) received or purchased at least one hermetic bag, but the number of bags obtained was typically not sufficient to store all harvested maize.

Self-selection into technology use thus limits the extent to which causal claims can be made about the impact of specific technologies. Nonetheless, examining the level of contamination in maize stored by farmers who only received drying sheets versus those who also used the maize dryer, hermetic storage bags, or both, provides suggestive evidence about the relative contribution of each technology to the overall reduction in aflatoxin achieved through the randomized intervention. Table 6 shows mean aflatoxin levels and 95% confidence intervals for farmers in the control group and within the treatment group by technology use. Because the number of observations per category is small, Panel A of Table 6 shows these figures within the treatment group by use of plastic sheets for sun drying (ignoring whether the mobile dryer or hermetic storage bags were used), Panel B presents the same statistics by category of dryer use (not taking into stated use of plastic sheets for sun drying, or whether the sampled maize was stored in a hermetic bag), and Panel C is broken down based on whether the sampled maize was stored in a hermetic bag, disregarding whether the mobile dryer or plastic sheets were used. Panel D presents results within the treatment group across the full breakdown of technology use categories.

**Table 6:** Comparison of aflatoxin contamination in stored home-grown maize, by use of technology: means ± standard error and confidence intervals.

	Mean aflatoxin (ppb) ± *SE*	95% *CI*	Observations
Control	18.5 ± 3.1	(12.5, 24.5)	98
*Panel A*			
Did not dry on plastic sheet	11.1 ± 5.3	(0.6, 21.5)	22
Dried on plastic sheet	7.3 ± 2.2	(2.9, 11.6)	51
*Panel B*			
Did not use mobile dryer	9.5 ± 2.8	(4.1, 14.9)	58
Used mobile dryer	4.1 ± 1.2	(1.7, 6.5)	15
*Panel C*			
Did not use hermetic storage bag	5.5 ± 1.5	(2.6, 8.5)	46
Used hermetic storage bag	13.3 ± 5.4	(2.3, 24.3)	27
*Panel D*			
Training only	10.9 ± 7.3	(-3.4, 25.3)	8
Training, and used plastic	3.9 ± 1.2	(1.5, 6.3)	28
Training, and used dryer	5.0 ± 1.6	(2.0, 8.1)	7
Training, and used plastic + dryer	7.5 ± 4.4	(-1.1, 16.1)	3
Training, and used hermetic storage	29.3 ± 26.1	(-21.8, 80.5)	4
Training, and used plastic + hermetic	13.2 ± 5.8	(1.9, 24.5)	18
Training, and used dryer + hermetic	1.1 ± 0.2	(0.6, 1.6)	3
Training, and used plastic + dryer + hermetic	0.6 ± 0.6	(-0.5, 1.7)	2

For both plastic and the drying service, mean aflatoxin is lower when the technology is used than when it is not used. Compared to maize stored by those in the control group using a *t*-test of means, dryer-dried maize (Panel B) contained 78% less aflatoxin (*N* = 113, *t* = 1.82 *P = *0.07). This is very close to the reduction reported by an experimental study by Kaaya and Kyamuhangire (2010) who tested a similar biomass-powered dryer, and found an 85% reduction after three months in storage compared to maize that had been dried on the bare ground. Maize that farmers reported had been dried on a plastic sheet was 61% less contaminated than that dried on another surface (*N* = 149, *t* = 2.47, *P* = 0.014). A study testing the impact of drying sheet distribution among Ghanaian groundnut farmers similarly found a 52% reduction in aflatoxin levels relative to those who were not given drying sheets (Hoffmann et al., 2017).

Maize sampled from hermetic bags also did not differ statistically in terms of aflatoxin contamination from that stored in traditional woven polypropylene bags, but the mean level in hermetically stored maize is higher. Recent experimental evidence shows similar levels of aflatoxin in maize stored in hermetic bags versus traditional woven bags when grain moisture content is above 14%, and far lower aflatoxin in hermetically stored bags when moisture content is below this level (Williams et al., 2014; Ng'ang'a et al., 2016). Hermetic storage is effective against Aspergillus, the fungal species that produce aflatoxin, because these require air to survive. However, anaerobic bacteria can thrive without oxygen as long as sufficient moisture is present. Grain losses may therefore occur if maize that has not been well dried is stored hermetically.

Splitting the categories further to show contamination under every combination of technology use, suggests that hermetic storage is very effective when maize is also dried down using the mobile dryer, but less so when maize is sun-dried. Those who used both the mobile dryer and hermetic storage had by far the lowest levels of aflatoxin, at 0.9 ppb on average. However, since there are only five observations in this category (even pooling across plastic use) differences in aflatoxin between this group and any other technology use category in the treatment group are indistinguishable from zero. Hermetic storage bags were given or sold only to farmers whose maize was found to have a mean moisture content of 13.5% or lower, regardless of dryer use. The results, however, suggest that despite this precaution, the effect of hermetic storage on sun-dried maize was muted. One potential explanation is that the use of a homogenized maize sample for moisture assessment may have masked within-bag variation, thus allowing maize above the recommended 13.5% moisture content to be stored hermetically; another possibility is that the maize stored hermetically was not the same as that for which moisture content was tested.

Another finding that emerges from Panel D is that training plus use of plastic sheeting alone appears to be highly effective. The farmers in this category had aflatoxin levels 79% lower than those in the control group (*N* = 126, *t* = 2.52 *P = *0.01). Even those farmers in treatment villages who did not use any of the technologies provided appear to have lower aflatoxin than those in the control group, though with only 8 farmers in this group, the difference is not statistically distinguishable from zero. Also of interest is whether farmers treat maize differently for own use versus for sale. By the time of maize sample collection, only 21 farmers had home-produced maize stored for consumption and sale separately: 11 in the control group and 10 in the treatment group. Among control group farmers, the level of contamination was higher in maize stored for sale at 16.1 ppb, versus 3.6 ppb in maize stored for own consumption, though the difference was not statistically significant based on a paired *t*-test (*N* = 11, *t* = 1.26, *P = *0.24). Among farmers in the treatment group, this gap closed to 6.2 ppb in maize for sale versus 4.1 ppb in maize for consumption (*N* = 10, *t* = 0.69, *P = *0.51).

## 4 Discussion

The primary contribution of this paper is to describe the impact of a package of an intervention designed to improve post-harvest practices on maize losses and aflatoxin contamination. The intervention, which consisted of a training session on aflatoxin and practices to prevent contamination, distribution of plastic sheets, and access to a mobile maize dryer and hermetic storage, reduced aflatoxin contamination by 53% three months after harvest. Farmers also reported significant reductions in losses to pests and mold. Our results on aflatoxin are comparable to those of Turner et al. (2005), who report a 59% reduction in the aflatoxin levels of stored groundnuts three months after harvest in Gambian villages where training on post-harvest practices, as well as woven drying mats, jute bags, pallets, and insecticides were provided.

Farmers who still had home-produced maize in store when samples were collected for aflatoxin analysis three months after harvest had higher socio-economic status on average than those who had already consumed or sold all of their maize. However, as shown in Table 7, the profiles of farmers within the treatment group who used plastic drying sheets and those who did not are similar, indicating that poorer farmers also benefited from the intervention. Moreover, the subset of households with stored maize are well-off only in relative terms; 21% of the heads of these households had completed secondary education, and the mean monthly consumption value per adult equivalent was 4240 KSh, equivalent to approximately $46 USD at the time.

**Table 7:** Comparison of household characteristics, by use of plastic sheets (treatment group only): means ± standard error, and test of the difference between means.

Variable	Mean ± *SE*, households that did not dry maize on plastic sheets provided	Mean ± *SE*, households that dried maize on plastic sheets provided	|*Z*-value| of means test	*P*-value of means test	Observations
Household size	5 ± 0.2	5.3 ± 0.2	1.77	(0.08)	212
Head completed secondary	0.14 ± 0.03	0.23 ± 0.04	1.63	(0.10)	212
Monthly consumption value/adult equivalent (KSh)	4083 ± 242	3691 ± 318	0.9	(0.37)	212
Number of non animal assets	1.9 ± 0.16	1.9 ± 0.18	0.18	(0.85)	212
Total maize harvest during both seasons (kg)	673 ± 95	670 ± 111	0.03	(0.97)	205
Sold any maize past year	0.40 ± 0.05	0.45 ± 0.05	0.72	(0.47)	209
Heard of aflatoxin	0.63 ± 0.05	0.66 ± 0.05	0.8	(0.43)	211
Drying maize well before storage prevents aflatoxin	0.88 ± 0.03	0.85 ± 0.04	0.51	(0.61)	211
Drying maize off bare ground prevents aflatoxin	0.26 ± 0.04	0.26 ± 0.04	0.1	(0.92)	211

Notes: Standard errors in means tests are bootstrapped and clustered at the village level cluster.

Non-experimental selection into technology use and small sample sizes preclude drawing strong conclusions about the relative contributions of training and the use of drying sheets, the mobile dryer, and hermetic storage to the overall impact. With this caveat in mind, the mean aflatoxin concentration was 79% lower in maize stored by farmers who had attended training and reported using a plastic sheet to sun-dry maize, but used neither the mobile dryer nor hermetic storage bags, compared to those assigned to the control group. This suggests that provision of drying sheets alone, an extremely simple and relatively low-cost technology at $6.70 per farmer including training costs, could achieve a substantial reduction in aflatoxin contamination in small-holder produced maize. Drying sheets cost approximately $5 per farmer; the $1.70 training cost assumes two meetings of 25 farmers each per day, a vehicle rental cost of $60, and a trainer wage of $25.

The cost-effectiveness of this strategy appears to compare favorably to other approaches for aflatoxin control. For example, the material cost of the aflatoxin biocontrol product, Aflasafe™ KE01 would be $8.40 for the average farmer in our study based on its current cost of $16 per hectare and mean land area under maize. This does not include the cost of training farmers to apply Aflasafe™ at the correct time and rate, which is likely to be substantial. The cost of post-harvest interventions tested by Turner et al., at $50 per farmer (Wu and Khlangwiset, 2010), is likewise far above that of training and plastic sheet provision. Experimental work to test the efficacy of drying sheets alone for aflatoxin control in settings similar to the one described in this paper would validate the suggestive evidence provided in this paper. As aflatoxin contamination varies widely from year to year, such research would ideally be performed over several seasons, and would also test the longevity of different drying sheet materials.

## Declarations of interest

None.

## Funding sources

Funding for this research was provided by the Ministry for Foreign Affairs of Finland through the FoodAfrica Programme (signed on April 10, 2012), UK aid from the British people (signed on January 16, 2012), the CGIAR Research Program on Agriculture for Nutrition and Health (A4NH) led by the International Food Policy Research Institute, and the Bill and Melinda Gates Foundation Grand Challenge Explorations Award (Lab-on-Mobile-Device, OPP1112728) to Mobile Assay Inc. (Don Cooper Ph.D., PI).

## Role of funding sources

None of the sponsors of the research were involved in the study design; in the collection, analysis or interpretation of data; in the writing of this manuscript or in the decision to submit the article for publication.
